# Isotretinoin and adverse neuropsychiatric outcomes: retrospective cohort study using routine data*

**DOI:** 10.1111/bjd.21049

**Published:** 2022-04-12

**Authors:** Tapio Paljarvi, Tess McPherson, Sierra Luciano, Kimmo Herttua, Seena Fazel

**Affiliations:** ^1^ Department of Psychiatry Oxford University, Warneford Hospital Oxford OX3 7JX UK; ^2^ Department of Forensic Psychiatry University of Eastern Finland, Niuvanniemi Hospital Kuopio Finland; ^3^ Department of Dermatology Oxford University Hospitals Oxford OX3 9DU UK; ^4^ TriNetX LLC 125 Cambridgepark Drive, Suite 500 Cambridge MA 02140 USA; ^5^ Department of Public Health University of Southern Denmark Degnevej 14 Esbjerg DK‐6705 Denmark; ^6^ Oxford Health NHS Foundation Trust, Warneford Hospital Oxford OX3 7JX UK

## Abstract

**Background:**

Severe neuropsychiatric outcomes have been reported in individuals exposed to isotretinoin, but the evidence is inconclusive and complicated by several methodological limitations.

**Objectives:**

To establish and quantify the association between isotretinoin use for acne and 1‐year incident neuropsychiatric adverse outcomes.

**Methods:**

A propensity score‐matched cohort study of electronic medical records between the years 2013 and 2019 with patients followed up for 1 year after their index dispensed prescription was conducted. The database included over 12 million patients aged 12–27 years. We analysed data for individuals with acne in this age range with a dispensed prescription for isotretinoin or a control prescription. Outcomes included diagnoses of any incident sleep or mental health disorder, or nonfatal self‐harm within 1 year of the index prescription.

**Results:**

We included 30 866 patients prescribed isotretinoin for their acne, 44 748 prescribed oral antibiotics, 108 367 prescribed topical anti‐acne agents and 78 666 patients with acne but without an anti‐acne prescription. After propensity score matching for baseline confounders, the odds ratio (OR) for any incident neuropsychiatric outcomes in patients with acne exposed to isotretinoin was 0·80 [95% confidence interval (CI) 0·74–0·87] compared with those on oral antibiotics; 0·94 (95% CI 0·87–1·02) compared with those using topical anti‐acne medicines; and 1·06 (95% CI 0·97–1·16) compared with those without a prescription for anti‐acne medicines. Patients exposed to isotretinoin experienced significantly more incident physical symptoms than patients in any of the three comparison cohorts.

**Conclusions:**

Isotretinoin was not independently associated with excess adverse neuropsychiatric outcomes at the population level. When monitoring potential adverse outcomes during isotretinoin treatment, clinicians should also consider the high mental health burden associated with treatment‐resistant acne and the potential contribution of physical side‐effects of prescribed medication on mental health.

## Graphical abstract

**What is already known about this topic?**

- Severe neuropsychiatric outcomes have been reported in individuals exposed to isotretinoin, but the evidence is inconclusive and complicated by several methodological limitations.
- The neuropsychiatric risks potentially associated with isotretinoin treatment are an ongoing concern to clinicians and individuals with treatment‐resistant acne.
- Current isotretinoin prescribing guidelines recommend close monitoring for adverse mental health outcomes during isotretinoin treatment.

**What does this study add?**

- After controlling for various potential sources of bias, isotretinoin was associated with a reduced incidence of adverse psychiatric outcomes (odds ratio 0·80, 95% confidence interval 0·74–0·87) compared with oral antibiotics.
- Isotretinoin appeared to reduce the excess psychiatric risk associated with treatment‐resistant moderate‐to‐severe acne.
- In monitoring potential adverse outcomes during isotretinoin treatment, clinicians should also consider the high mental health burden associated with treatment‐resistant acne and the potential contribution of physical side‐effects of the prescribed medication on mental health.

In this propensity score matched cohort study of electronic medical records, we found that after controlling for various potential sources of bias, isotretinoin was associated with a reduced incidence of adverse psychiatric outcomes (odds ratio 0.80, 95% confidence interval 0.74–0.87) when compared to oral antibiotics. Isotretinoin thus appeared to reduce the excess psychiatric risk associated with treatment‐resistant moderate‐to‐severe acne.

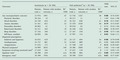

**Linked Comment:** J. Ravencroft and L. Eichenfield. Br J Dermatol 2022; 187:8–9.

Plain language summary available online

Acne vulgaris (acne) is a highly prevalent long‐term inflammatory skin condition with symptom onset typically in adolescence, with the highest prevalence between the ages of 14 and 19 years.^1^, ^2^, ^3^ Acne is known to affect both physical and psychosocial health and wellbeing.^4^ Oral isotretinoin is a vitamin A derivative, and it is indicated for the treatment of recalcitrant inflammatory acne. Isotretinoin can also be used off‐label, including to treat and prevent certain types of skin cancer.^5^ Many patients treated with isotretinoin experience adverse side‐effects such as dry skin or mouth, headache or musculoskeletal pain.^6^, ^7^ The various mucocutaneous and central nervous system side‐effects of isotretinoin are mostly dose related and some, such as dry skin and skin irritation, are common with lower therapeutic doses. Some patients also experience an initial worsening of acne symptoms (flare‐up) after the start of treatment.^8^ In addition, severe neuropsychiatric outcomes, such as anxiety, depression, suicidal ideation, self‐harm and completed suicide, have been reported. However, the evidence base for severe neuropsychiatric outcomes is mixed and inconclusive.^9^, ^10^, ^11^, ^12^, ^13^, ^14^, ^15^, ^16^, ^17^, ^18^, ^19^, ^20^, ^21^, ^22^ For example, isotretinoin has been associated with both increased^14^ and reduced^16^ risk of depression.

There are several methodological limitations in the current evidence about the association between isotretinoin and severe neuropsychiatric adverse outcomes.^14^ Much of the evidence is based on small cohorts of patients leading to a lack of precision in effect estimates. For example, a recent meta‐analysis of 31 studies was able to include only 1411 patients with an assessment of depression before and after isotretinoin exposure.^16^ More importantly, because acne severity in those patients exposed to isotretinoin and patients in the unexposed control groups have likely differed, confounding by indication explains part of the conflicting evidence. To address confounding by indication in relation to isotretinoin‐associated adverse neuropsychiatric outcomes, we conducted a propensity score‐matched retrospective cohort study to compare the incidence of neuropsychiatric outcomes in patients exposed to isotretinoin and patients receiving other types of acne treatment by using information from the electronic medical records of over 12 million patients aged 12–27 years from 56 healthcare organizations. We defined three different patient populations as control groups for the patients who had been exposed to isotretinoin to indirectly control for the severity of acne.

## Patients and methods

We used the TriNetX (TriNetX LLC, Cambridge, MA 02140, USA) proprietary patient repository and analytics platform to identify patients with acne. The TriNetX Analytics Network provided access to pseudonymized aggregate‐level information on electronic healthcare records (EHRs, including diagnoses and dispensed prescription medicines) from 56 healthcare organizations mainly in the USA (91% of patients in the USA and 9% in Europe, Latin America and Asia‐Pacific). As this study used only de‐identified patient records and did not involve the collection, use or transmittal of individually identifiable data, this study was exempted from institutional review board approval. (See Appendix S1 in the Supporting Information for more details on the TriNetX Analytics Network.)

Data for this study were accessed via the TriNetX platform and analysed in August 2021 by using the TriNetX built‐in query builder. All data processing was conducted using the TriNetX built‐in proprietary algorithms. All diagnoses were identified using the International Classification of Diseases, tenth revision, clinical modification (ICD10‐CM) codes, and dispensed prescription medicines were identified using the RxNorm codes (Table S1; see Supporting Information).

### Design

The TriNetX Analytics Network included over 12 million patients (*n* = 12 371 366) aged 12–27 years between the years 2013 and 2019. Patients were eligible if they were aged 12–27 years at index prescription, had diagnosed acne, did not have diagnosed neoplasms, and were alive for the subsequent 12 months after the index prescription. We focused on this age group because in the USA, isotretinoin is indicated in patients aged 12 or older and because 88% (30 866/35 100) of the eligible patients with acne treated with isotretinoin were within this age range. Excluding patients with acne aged over 27 years also limits the prevalence of adult‐onset acne and various comorbidities that could confound the observed association in older patients.

After excluding patients with a history of neoplasms in their EHR and patients who died during the study period, 382 340 patients had a diagnosis of acne in their EHR; of which, 78% had a diagnosis for acne vulgaris. Of these patients with acne, 30 866 had at least one dispensed prescription for isotretinoin and were thus included in the isotretinoin‐exposed cohort.

To indirectly capture the severity of acne symptoms, we defined three different patient populations as control cohorts for the isotretinoin treatment cohort. First, 44 748 patients were included in the oral antibiotics (erythromycins/macrolides or tetracyclines) cohort of patients who did not have dispensed prescriptions for isotretinoin. Second, 108 367 patients were included in the topical anti‐acne cohort of patients who did not have dispensed prescriptions for isotretinoin or for the selected oral antibiotics (erythromycins or tetracyclines). Third, 78 666 patients were included in the acne control cohort of patients who did not have dispensed prescriptions for isotretinoin, any topical anti‐acne agents or for the selected oral antibiotics. For isotretinoin, oral antibiotics and topical anti‐acne agents, we used the first dispensed prescription in the data as the index prescription; whereas for the third control cohort we used the first healthcare visit because of acne as the index event.

Propensity score matching was used to improve comparability between the isotretinoin‐exposed and control (isotretinoin‐unexposed) cohorts in relation to the type of acne, comorbidities and history of mental health problems, that is, to control for confounders that could bias the comparisons. For propensity score matching, we used the TriNetX built‐in algorithm, which was based on 1: 1 nearest neighbour matching with a caliper of 0.1 SDs. We included covariates occurring any time before the index prescription or event, and the same set of covariates were used to match isotretinoin‐exposed cohort and the three unexposed control cohorts.

Cohorts were balanced at baseline for the following covariates: age at index prescription or event; sex; ethnicity/race; mental and behavioural disorders because of psychoactive substance use; schizophrenia, schizotypal, delusional and other non‐mood psychotic disorders; mood (affective) disorders; anxiety, dissociative, stress‐related, somatoform and other nonpsychotic mental disorders; sleep disorders not because of a substance or known physiological condition; disorders of adult personality and behaviour; behavioural and emotional disorders with onset usually occurring in childhood and adolescence; sleep disorders; vasomotor and allergic rhinitis; asthma; dermatitis and eczema; acne vulgaris; acne conglobata; other acne; unspecified acne; suicidal ideations; suicide attempt; intentional self‐harm; and dispensed prescriptions for sedatives and hypnotics; antidepressants; antipsychotics; and glucocorticoids. Patients in the isotretinoin cohort were successfully matched with patients in the oral antibiotics cohort (92%, 28398/30866), in the topical anti‐acne agents cohort (99%, 30 435/30 866) and in the acne control cohort without dispensed prescriptions for the selected acne medications (92%, 28 417/30 866).

Tables S2–S4 (see Supporting Information) show the distributions of the patient characteristics before and after matching at baseline for the isotretinoin and for the three control cohorts. After matching, the cohorts were balanced for all the covariates included, that is, all standard differences were < 0·1 after propensity score matching.

### Outcome measurement

Primary outcome measures were 12‐month incident main categories of neuropsychiatric diagnoses, including psychotic disorders; mood disorders; anxiety, dissociative, stress‐related, somatoform and other nonpsychotic mental disorders; adult personality and behaviour disorders; behavioural and emotional disorders with onset usually occurring in childhood and adolescence; sleep disorders; and nonfatal self‐harm, which also included events of undetermined intent. We also included an incident outcome for any of the above neuropsychiatric conditions.

Secondary outcomes were used to triangulate the primary outcomes and were used as proxies for clinically important neuropsychiatric outcomes receiving treatment. These secondary outcomes were 12‐month incident dispensed prescriptions for psychotropic medicines, including sedatives and hypnotics; antidepressants; and antipsychotics.

Additionally, we included various physical symptoms of common side‐effects associated with isotretinoin treatment: headache, pain, eye conditions, conditions of mouth and lips, nausea and vomiting, malaise and fatigue. This is not an exhaustive list of all known side‐effects, but these reflect some of the most common physical side‐effects of isotretinoin. We also included an outcome for potential mental health side‐effects not fulfilling diagnostic criteria for any of the primary diagnoses, that is, symptoms and signs involving emotional state. This diagnostic group includes codes for symptoms such as nervousness, restlessness and agitation, unhappiness, demoralization and apathy, irritability and anger, hostility, violent behaviour, state of emotional shock and stress, low self‐esteem, worries, excessive crying, anhedonia, homicidal and suicidal ideations, emotional lability and impulsiveness. Furthermore, 12‐month inpatient and emergency visits were included as proxy outcomes for general health status during follow‐up.

We also examined the association between acne symptom severity and adverse neuropsychiatric outcomes independently of isotretinoin, that is, we included comparisons between the three control cohorts. In these models, we compared patients with dispensed oral antibiotics (erythromycins or tetracyclines) who were not exposed to isotretinoin and patients with dispensed topical anti‐acne agents who were not exposed to isotretinoin or oral antibiotics (erythromycins or tetracyclines). We also compared patients with dispensed topical anti‐acne agents who were not exposed to isotretinoin or oral antibiotics (erythromycins or tetracyclines) and patients who did not have dispensed prescriptions for any of the selected acne medications.

We included only incident outcomes for the primary and secondary outcomes. That is, in each model, we included only patients who did not have a recorded history of the given outcome in their EHR. We used a 14‐day washout period after the index prescription for measuring outcomes to reduce bias from conditions already present at the time of the index prescription. The EHR data included information on whether the patient had died while in hospital but did not include information on the cause of death. Those who died as inpatients were excluded from the main analyses. As a result of the lack of cause of death information we were unable to establish whether these deaths could be attributable to isotretinoin treatment. The number of inpatient deaths during the 12‐month follow‐up period are reported in Table S5 (see Supporting Information) for patients with acne treated with isotretinoin and those where acne was treated with oral antibiotics. For all 12‐month incident outcomes, we calculated incidence rates per 1000 person‐years and odds ratios (ORs) with 95% confidence intervals (95% CI). Patients with missing information were excluded from the analyses.

## Results

Before propensity score matching, the distributions of baseline patient characteristics differed across the isotretinoin cohort and the three control cohorts (Tables S2–S4, see Supporting Information). The largest differences were seen between patients with acne in the isotretinoin cohort and patients in the topical anti‐acne agents cohort (Table S3), whereas, the differences were smallest when patients in the isotretinoin cohort were compared with patients in the oral antibiotics cohort (Table S4). After matching, the mean age at index prescription was 18 years (SD 4), about half of the patients were women, about three‐quarters were white, and almost all patients had a diagnosis of acne vulgaris in their medical records.

Without controlling for acne severity by using information on the different types of treatment, acne diagnosis was associated with an increased odds of 1‐year incident adverse neuropsychiatric outcomes (OR 1·46, 95% CI 1·43–1·50), when compared with the sex‐, age‐ and race‐matched general patient population without an acne diagnosis (Table S6).

After matching for all included covariates, patients with dispensed prescriptions for any topical anti‐acne agents had an increased odds for incident adverse neuropsychiatric outcomes (OR 1·16, 95% CI 1·09–1·22), compared with patients with an acne diagnosis but who did not have dispensed prescriptions for any of the selected acne medication (Table S7). Similarly, patients with acne taking oral antibiotics had an increased odds for incident adverse neuropsychiatric outcomes (OR 1·19, 95% CI 1·11–1·27) compared with patients with acne using topical anti‐acne agents (Table S8). These results showed that the cohorts of patients on oral antibiotics and using topical anti‐acne agents captured quantifiable differences in the underlying risk for adverse neuropsychiatric outcomes independently of exposure to isotretinoin.

### Inpatient mortality

No clear difference in inpatient mortality between patients with acne exposed to isotretinoin and patients with acne prescribed oral antibiotics was found during the 12‐month follow‐up period (Table S5). The statistically nonsignificant ORs suggested a small excess in inpatient mortality among those exposed to isotretinoin compared with those exposed to oral antibiotics at 9–12 months after the index prescription. However, the wide 95% CIs indicated a high level of uncertainty in the ORs because of the small number of inpatient deaths. Of the 19 patients who died in hospital during the 12‐month follow‐up in the isotretinoin cohort, none had healthcare contacts because of self‐harm during the follow‐up.

### Isotretinoin exposure

The OR for any incident neuropsychiatric outcomes in patients exposed to isotretinoin was 1·06 (95% CI 0·97–1·16) compared with patients with an acne diagnosis but who did not have dispensed prescriptions for any of the selected acne medication (Table 1); 0·94 (95% CI 0·87–1·02) compared with patients using topical anti‐acne medicines (Table 2); and 0·80 (95% CI 0·74–0·87) compared with patients on oral antibiotics (Table 3).

**Table 1:** One‐year incidence of neuropsychiatric outcomes among patients with acne exposed to isotretinoin compared with patients with acne who were not exposed to isotretinoin, oral antibiotics for acne or topical anti‐acne agents during follow‐up^a^

Outcomes	Isotretinoin (*n* = 28 417)			No selected anti‐acne medication^b^ (*n* = 28 417)			Odds ratio	95% CI
	Patients, total, *n*	Patients with incident outcome, *n*	IR/1000	Patients, total, *n*	Patients with incident outcome, *n*	IR/1000		
Any neuropsychiatric outcome	23 536	1064	45	23 438	998	42	1·06	0·97–1·16
Psychotic disorders	28 357	23	1	28 364	19	1	1·21	0·66–2·22
Mood disorders	26 447	600	23	26 608	536	20	**1**·**13**	1·00–1·27
Anxiety disorders	25 689	771	30	25 978	692	27	**1**·**13**	1·02–1·25
Personality disorders	28 070	53	2	28 106	61	2	0·87	0·60–1·26
Behavioural disorders	26 403	254	10	26 585	277	10	0·92	0·78–1·09
Sleep disorders	27 440	317	11	27 507	268	10	**1**·**19**	1·10–1·40
Self‐harm, nonfatal	28 067	100	3	28 061	80	3	1·25	0·93–1·68
Dispensed prescriptions								
Sedatives and hypnotics	26 515	591	22	26 607	415	15	**1**·**44**	1·27–1·63
Antidepressants	25 604	815	32	25 753	455	18	**1**·**83**	1·63–2·05
Antipsychotics	27 857	161	6	27 892	106	4	**1**·**52**	1·19–1·95
Physical symptoms^c^	19 593	3081	157	20 287	1720	85	**2**·**01**	1·89–2·14
Symptoms involving emotional state^d^	27 760	226	8	27 858	162	6	**1**·**40**	1·15–1·72
Inpatient visits^e^	28 417	610	n/a	28 417	401	n/a	**1**·**53**	1·35–1·74
Emergency visits^e^	28 417	1838	n/a	28 417	1360	n/a	**1**·**38**	1·28–1·48

CI, confidence interval; IR, incidence rate per 1000 person‐years; n/a, not applicable. ^a^Patients aged 12–27 years at index prescription (isotretinoin) or index visit (acne control group) in 2013– 2019 after propensity score matching for all covariates. Oral antibiotics: erythromycins(macrolides) or tetracyclines. Results in bold are significant. ^b^Healthcare contact during which a diagnosis for acne was recorded. ^c^Physical symptoms of common isotretinoin side‐effects, such as headache, nausea and vomiting, dry mouth, conjunctivitis, pain, cheilitis and fatigue. ^d^Symptoms and signs involving emotional state, including various diagnoses for agitation and aggression. ^e^Including patients with visits before index prescription (before start of follow‐up).

**Table 2:** One‐year incidence of neuropsychiatric outcomes among patients with acne exposed to isotretinoin compared with patients with dispensed prescriptions for topical anti‐acne agents who were not exposed to isotretinoin or oral antibiotics for acne during follow‐up^a^

Outcomes	Isotretinoin (*n* = 30 435)			Topical anti‐acne agents^b^ (*n* = 30 435)			Odds ratio	95% CI
	Patients, total, *n*	Patients with incident outcome, *n*	IR/1000	Patients, total, *n*	Patients with incident outcome, *n*	IR/1000		
Any neuropsychiatric outcome	24 977	1172	47	24 596	1220	50	0·94	0·87–1·02
Psychotic disorders	30 372	23	1	30 355	23	1	1·00	0·56–1·78
Mood disorders	28 258	688	24	28 278	724	26	0·95	0·85–1·06
Anxiety disorders	27 362	862	31	27 412	942	34	0·91	0·83–1·00
Personality disorders	30 035	60	2	30 030	80	3	0·75	0·54–1·05
Behavioural disorders	28 251	285	10	28 403	324	11	0·88	0·75–1·04
Sleep disorders	29 286	359	12	29 299	326	11	1·10	0·95–1·28
Self‐harm, nonfatal	30 034	118	4	29 992	97	3	1·22	0·93–1·59
Dispensed prescriptions								
Sedatives and hypnotics	27 967	635	23	27 895	639	23	0·99	0·89–1·11
Antidepressants	26 774	851	32	26 766	738	27	**1**·**16**	1·05–1·28
Antipsychotics	29 703	181	6	29 726	162	5	1·12	0·90–1·38
Physical symptoms^c^	20 566	3263	159	20 646	1922	93	**1**·**84**	1·73–1·95
Symptoms involving emotional state^d^	29 690	260	9	29 665	215	7	**1**·**21**	1·10–1·45
Inpatient visits^e^	30 435	669	n/a	30 435	705	n/a	0·95	0·85–1·05
Emergency visits^e^	30 435	2037	n/a	30 435	2007	n/a	1·02	0·95–1·08

CI, confidence interval; IR, incidence rate per 1000 person‐years; n/a, not applicable. ^a^Patients aged 12–27 years at index prescription in 2013–2019 after propensity score matching for all covariates. Oral antibiotics: erythromycins(macrolides) or tetracyclines. Results in bold are significant. ^b^Topical anti‐acne agents, such as topical antibiotic preparations, tretinoin and benzoyl peroxide. ^c^Physical symptoms of common isotretinoin side‐effects, such as headache, nausea and vomiting, dry mouth, conjunctivitis, pain, cheilitis and fatigue. ^d^Symptoms and signs involving emotional state, including various diagnoses for agitation and aggression. ^e^Including patients with visits before index prescription (before start of follow‐up).

**Table 3:** One‐year incidence of neuropsychiatric outcomes among patients with acne exposed to isotretinoin compared with patients with dispensed prescriptions for oral antibiotics for acne who were not exposed to isotretinoin during follow‐up^a^

Outcomes	Isotretinoin (*n* = 28 398)			Oral antibiotics^b^ (*n* = 28 398)			Odds ratio	95% CI
	Patients, total, *n*	Patients with incident outcome, *n*	IR/1000	Patients, total, *n*	Patients with incident outcome, *n*	IR/1000		
Any neuropsychiatric outcome	22 974	1091	47	22 756	1332	58	**0**·**80**	0·74–0·87
Psychotic disorders	28 334	23	1	28 317	21	1	1·09	0·61–1·98
Mood disorders	26 220	654	25	26 232	828	31	**0**·**78**	0·71–0·87
Anxiety disorders	25 341	822	32	25 370	1028	40	**0**·**79**	0·72–0·87
Personality disorders	28 003	61	2	28 018	82	3	0·74	0·53–1·04
Behavioural disorders	26 221	263	10	26 340	320	12	**0**·**82**	0·70–0·97
Sleep disorders	27 256	340	12	27 267	378	14	**0**·**90**	0·77–1·04
Self‐harm, nonfatal	28 003	106	4	27 997	124	4	**0**·**85**	0·66–1·11
Dispensed prescriptions								
Sedatives and hypnotics	26 025	610	23	26 127	694	26	**0**·**88**	0·79–0·98
Antidepressants	24 757	801	32	24 842	889	35	0·90	0·82–0·99
Antipsychotics	27 676	172	6	27 708	239	9	**0**·**72**	0·59–0·87
Physical symptoms^c^	18 899	3033	160	18 386	2068	112	**1**·**51**	1·42–1·60
Symptoms involving emotional state^d^	27 659	244	9	27 657	218	8	1·12	0·93–1·35
Inpatient visits^e^	28 398	650	n/a	28 398	804	n/a	**0**·**80**	0·72–0·89
Emergency visits^e^	28 398	1956	n/a	28 398	2359	n/a	**0**·**82**	0·77–0·87

CI, confidence interval; IR, incidence rate per 1000 person‐years; n/a, not applicable. ^a^Patients aged 12–27 years at index prescription in 2013–2019 after propensity score matching for all covariates. Results in bold are significant. ^b^Oral antibiotics: erythromycins(macrolides) or tetracyclines. ^c^Physical symptoms of common isotretinoin side‐effects, such as headache, nausea and vomiting, dry mouth, conjunctivitis, pain, cheilitis and fatigue. ^d^Symptoms and signs involving emotional state, including various diagnoses for agitation and aggression. ^e^Including patients with visits before index prescription (before start of follow‐up).

Compared with patients with acne who did not have dispensed prescriptions for any of the selected acne medications, patients in the isotretinoin cohort had increased odds for incident mood disorders, anxiety disorders and sleep disorders (Table 1). The strongest associations were observed for incident prescriptions for psychotropic medicines, such as for antidepressants (OR 1·83, 95% CI 1·63–2·05). An association was also observed for symptoms involving emotional state (OR 1·40, 95% CI 1·15–1·72).

Compared with patients with acne using topical anti‐acne agents, patients in the isotretinoin cohort had increased odds for incident prescriptions for antidepressants (OR 1·16, 95% CI 1·05–1·28) and for symptoms involving emotional state (OR 1·21, 95% CI 1·10–1·45), but all other ORs were statistically nonsignificant, and most were close to a null association (Table 2). There was a nonsignificantly increased odds for nonfatal self‐harm of 1·22 (95% CI 0·93–1·59), and the difference in incidence rate was 1/1000 person‐years (Table 2).

In contrast, compared with patients in the oral antibiotics cohort, the patients in the isotretinoin cohort had decreased odds for incident neuropsychiatric outcomes (OR 0·80, 95% CI 0·74–0·87), and this applied to all neuropsychiatric outcomes except for psychotic disorders and symptoms involving emotional state with statistically nonsignificant ORs (Table 3). The decreased odds were statistically significant for mood disorders, anxiety disorders, behavioural disorders and incident prescriptions for psychotropic medicines. There was no clear association for nonfatal self‐harm (OR 0·85, 95% CI 0·66–1·11).

Patients in the isotretinoin cohort experienced significantly more incident physical symptoms compared with any of the three comparison cohorts. The highest OR (2·01, 95% CI 1·89–2·14) for incident physical symptoms was observed when comparing patients in the isotretinoin‐exposed cohort with those without any of the selected acne medications (Table 1). Indicators for general health status (inpatient and emergency visits) showed a comparable pattern of associations with what was observed for mental health‐specific outcomes by the type of acne treatment (Tables 1, 2, 3).

## Discussion

We examined the association between isotretinoin and incident adverse neuropsychiatric outcomes over a year in 30 866 individuals aged 12–27 years at index prescription. We observed a consistent association between increasing acne severity as indicated by anti‐acne treatment options and incidence of adverse neuropsychiatric outcomes, but the findings showed that isotretinoin exposure did not add to the risk of neuropsychiatric adverse outcomes over and above what was associated with oral antibiotics. Instead, we observed that isotretinoin was associated with reduced incidence of anxiety, depression, sleep problems, nonfatal self‐harm, and prescriptions for psychotropic medicines, when compared with patients with acne who were propensity score matched and prescribed oral antibiotics. Isotretinoin treatment, therefore, appeared to mitigate the excess neuropsychiatric risk associated with recalcitrant moderate‐to‐severe acne.

Our results add to the evidence that has suggested improvement in symptoms of anxiety^17^ and depression^16^, ^20^ in individuals treated with isotretinoin. A recent observational study also found reduced symptoms of depression in patients treated with isotretinoin than in patients prescribed oral antibiotics.^23^ Compared with patients with acne who had prescriptions for topical anti‐acne agents, presumably for less severe acne, we observed a statistically nonsignificant association for nonfatal self‐harm among patients treated with isotretinoin (one excess case per 1000 person‐years, OR 1·22, 95% CI 0·93–1·59). However, a contrasting, statistically nonsignificant association was observed when the isotretinoin‐exposed cohort were compared with those with prescriptions for oral antibiotics (OR 0·85, 95% CI 0·66–1·11). This finding is also in line with previous observational research concluding that isotretinoin is unlikely to be independently associated with self‐harm at population level.^21^, ^24^ Furthermore, our finding that patients treated with oral antibiotics had a significantly increased odds for nonfatal self‐harm compared with those prescribed topical anti‐acne agents (OR 1·24, 95% CI 1·03–1·51) is consistent with previous work showing that the risk of self‐harm was already increased in patients with acne before isotretinoin treatment.^25^ Accumulating evidence, therefore, does not support the conclusion that isotretinoin would be independently associated with excess adverse neuropsychiatric outcomes at the population level.^16^, ^17^, ^18^, ^20^

Despite concerns over isotretinoin‐induced idiosyncratic adverse effects,^14^, ^18^ research aiming to identify potentially vulnerable patients is lacking. Being exposed to large amounts of vitamin A is known to have neurotoxic effects, but the ability of the therapeutic doses of isotretinoin typically used in treating acne to induce severe acute psychiatric symptoms has not been established. This could be partly because the focus has been on the direct effects of isotretinoin on psychiatric outcomes, such as on isotretinoin‐induced primary depression. Interaction between acne‐related symptoms and the relatively common physical and neurological side‐effects of isotretinoin has rarely been examined as a potential mechanism for idiosyncratic neuropsychiatric adverse outcomes associated with isotretinoin. For example, a bidirectional association between headache and depression has been established,^26^, ^27^ and co‐occurring headache and depression has also been reported among patients treated with isotretinoin.^28^

We observed that patients treated with isotretinoin significantly more often experienced incident physical symptoms than patients with acne receiving other types of treatment or no treatment with acne prescription medicines. An increased graded association by the severity of acne was observed for these physical symptoms. Acne‐related psychosocial stress in general or pre‐existing subclinical mental health problems together with an initial acne flare‐up or other isotretinoin‐induced physical side‐effects may trigger clinically relevant mental health symptoms in patients already vulnerable because of their severe acne, particularly among patients with limited coping and emotional resilience.^29^ Research on stressful life events has shown that even relatively minor and low‐intensity life events and experiences can trigger clinically relevant symptoms of anxiety and depression in vulnerable individuals.^30^ Similar mechanisms of underlying sustained and accumulated acne‐related psychological burden may contribute to an increased vulnerability of clinically relevant mental health problems among patients who experience added psychological burden because of acute isotretinoin‐induced physical or neurological side‐effects. Therefore, it can be suggested that psychosocial vulnerability in general, pre‐existing subclinical mental health problems, physical comorbidity, subjective and objective burden of acne symptoms, age at onset and duration of acne symptoms, and the frequency and intensity of isotretinoin‐induced physical and neurological side‐effects, all contribute to the risk of adverse mental health outcomes during isotretinoin treatment. In practice, therefore, the physical side‐effects of the medication should also be considered during isotretinoin treatment; and if appropriate, the dose reduced, particularly in patients with mental health symptoms. Future observational research should aim to establish the potential role of common physical and neurological side‐effects as mediators for adverse mental health outcomes during isotretinoin treatment.

As we used retrospective EHR data, we did not have control over treatment allocation. Our results thus represent real‐world treatment decisions. We did not have information on the duration of treatment or adherence, which may have confounded the observed associations. Among patients in a real‐world setting, there might be a considerable delay in seeking care for neuropsychiatric symptoms, particularly if symptoms are mild initially, which may obscure the correct sequence of events. We aimed to control for this by using a 14‐day washout period after index prescription for measuring incident outcomes to reduce potential bias from conditions that were already present at the time of index prescription. By propensity score matching, we aimed to control for confounding by indication and potential differences in baseline health status. However, it is possible that some residual confounding by indication remained in the models by unmeasured risk factors not captured by the EHR data.^31^

We were able to include a large number of patients from various healthcare organizations, but these organizations cannot be considered as representative of all healthcare organizations, and therefore differences in factors such as patient characteristics, data quality and isotretinoin prescription preferences across organizations may have affected the results. Also, if patients received treatment from healthcare organizations not participating in the TriNetX Network, before or after index prescription, this may have introduced bias because of misclassification in exposure status or loss to follow‐up for the outcomes. Furthermore, some of the isotretinoin‐induced physical or neurological side‐effects may have been misclassified, such as depression, because of unspecific or overlapping symptoms, such as fatigue, general malaise, emotional lability, and anhedonia.

We observed that patients prescribed with isotretinoin had a slightly higher incidence of symptoms and signs involving emotional state, which is a diagnostically heterogeneous and nonspecific group of mental health symptoms. Misclassification bias may be more likely among patients treated with isotretinoin because of enhanced awareness of potential isotretinoin‐induced side‐effects. This type of misclassification would have led to overestimating the association between isotretinoin and adverse neuropsychiatric outcomes. Patients prescribed with isotretinoin are regularly monitored for abnormal blood test results, meaning that these patients are regularly in contact with healthcare organizations. This may have further overestimated the incidence of neuropsychiatric outcomes among patients prescribed with isotretinoin. As we used EHRs to identify adverse neuropsychiatric outcomes, we only identified patients receiving treatment for these conditions. This will have underestimated the number of individuals experiencing these outcomes with less severe symptoms.

We did not have information on mortality, except for inpatient deaths. Further research with similarly improved design is thus needed to establish the association between isotretinoin and cause‐specific mortality. A major strength of our data was the relatively large number of patients initially prescribed isotretinoin, which improved the precision of our effect estimates. Furthermore, we were able to establish the associations for relatively rare outcomes, such as psychotic disorders, sleep disorders, personality disorders, behavioural disorders, and nonfatal self‐harm.

In conclusion, when indirectly controlling for severity of acne, we found that isotretinoin was not independently associated with adverse neuropsychiatric outcomes at the population level. Patients with acne with prescriptions for oral antibiotics, but without prescriptions for isotretinoin, had the highest incidence of adverse neuropsychiatric outcomes over the 1‐year follow‐up period. These patients also had an increased odds for nonfatal self‐harm compared with those with less severe acne. This finding further highlights the need for timely and effective acne management.

## Author contributions

**Tapio Paljarvi:** Conceptualization (equal); formal analysis (lead); investigation (equal); methodology (equal); validation (equal); writing – original draft (lead); writing – review and editing (equal). **Tess McPherson:** Conceptualization (equal); methodology (equal); supervision (equal); writing – review and editing (equal). **Sierra Luciano:** Data curation (lead); formal analysis (supporting); investigation (equal); methodology (supporting); software (lead); validation (equal); writing – review and editing (equal). **Kimmo Herttua:** Conceptualization (equal); methodology (equal); writing – review and editing (equal). **Seena Fazel:** Conceptualization (equal); investigation (equal); resources (lead); supervision (lead); writing – review and editing (equal).

## Supporting information


**Appendix S1** Additional methods.
**Table S1** Medical codes used in defining the cohorts, in propensity score matching and in defining the outcomes.
**Table S2** Baseline characteristics of patients with acne in the isotretinoin and in the acne control (not exposed to any anti‐acne agents, or oral antibiotics for acne) cohorts before and after matching.
**Table S3** Baseline characteristics of patients with acne in the isotretinoin and in the topical anti‐acne agents cohorts before and after matching.
**Table S4** Baseline characteristics of patients with acne in the isotretinoin and in the oral antibiotics cohorts before and after matching.
**Table S5** Overall inpatient mortality among patients with acne exposed to isotretinoin compared with patient with dispensed prescriptions for oral antibiotics for acne who were not exposed to isotretinoin during follow‐up by time after the index prescription.
**Table S6** One‐year incidence of neuropsychiatric outcomes among patients with acne compared with patients who were not diagnosed with acne.
**Table S7** One‐year incidence of neuropsychiatric outcomes among patients with acne with dispensed prescriptions for topical anti‐acne agents who were not exposed to isotretinoin or oral antibiotics for acne compared with patients with acne who were not exposed to isotretinoin, oral antibiotics for acne or topical anti‐acne agents during follow‐up.
**Table S8** One‐year incidence of neuropsychiatric outcomes among patients with acne with dispensed prescriptions for oral antibiotics for acne who were not exposed to isotretinoin compared with patients with acne with dispensed prescriptions for topical anti‐acne agents who were not exposed to isotretinoin or oral antibiotics for acne during follow‐up.

**Video S1** Author video.
