# Supplementary material for: Isotretinoin and adverse neuropsychiatric outcomes: retrospective cohort study using routine data
Source: Br J Dermatol. 2022 Apr 12;187(1):64–72. doi: 10.1111/bjd.21049 (PMC9543533; doi:10.1111/bjd.21049)
Supplement: Supplementary file 1 — Appendix S1 Additional methods. Table S1 Medical codes used in defining the cohorts, in propensity score matching and in defining the outcomes. Table S2 Baseline characteristics of patients with acne in the isotretinoin and in the acne control (not exposed to any anti‐acne agents, or oral antibiotics for acne) cohorts before and after matching. Table S3 Baseline characteristics of patients with acne in the isotretinoin and in the topical anti‐acne agents cohorts before and after matching. Table S4 Baseline characteristics of patients with acne in the isotretinoin and in the oral antibiotics cohorts before and after matching. Table S5 Overall inpatient mortality among patients with acne exposed to isotretinoin compared with patient with dispensed prescriptions for oral antibiotics for acne who were not exposed to isotretinoin during follow‐up by time after the index prescription. Table S6 One‐year incidence of neuropsychiatric outcomes among patients with acne compared with patients who were not diagnosed with acne. Table S7 One‐year incidence of neuropsychiatric outcomes among patients with acne with dispensed prescriptions for topical anti‐acne agents who were not exposed to isotretinoin or oral antibiotics for acne compared with patients with acne who were not exposed to isotretinoin, oral antibiotics for acne or topical anti‐acne agents during follow‐up. Table S8 One‐year incidence of neuropsychiatric outcomes among patients with acne with dispensed prescriptions for oral antibiotics for acne who were not exposed to isotretinoin compared with patients with acne with dispensed prescriptions for topical anti‐acne agents who were not exposed to isotretinoin or oral antibiotics for acne during follow‐up. [file BJD-187-64-s002.docx]

**Supporting Information**

**Appendix S1: Methods**

Data

The HCOs included in the TriNetX Analytics Network are typically large academic medical centres that provide a range of healthcare services, including emergency, outpatient, and inpatient care. A single HCO typically has more than one facility, and EHR data from all these facilities are available on the TriNetX platform. Patients are included regardless of insurance status. The quality of TriNetX data is ensured by and evaluated against pre-specified quality standards.^1^

TriNetX, LLC is compliant with the Health Insurance Portability and Accountability Act (HIPAA), the US federal law which protects the privacy and security of healthcare data, and any additional data privacy regulations applicable to the contributing HCO. TriNetX is certified to the ISO 27001:2013 standard and maintains an Information Security Management System (to ensure the protection of the healthcare data it has access to. Any data displayed on the TriNetX Platform in aggregate form only contains de-identified data as determined by a qualified expert as defined in Section §164.514(b)(1) of the HIPAA Privacy Rule.

The TriNetX data has been used for epidemiological research on various neuropsychiatric outcomes.^2-6^

**References**

1. Topaloglu U, Palchuk MB. Using a Federated Network of Real-World Data to Optimize Clinical Trials Operations. JCO Clin Cancer Inform. 2018 Dec;2:1-10. doi: 10.1200/CCI.17.00067.
2. Paljarvi T, Strang J, Quinn PD, Luciano S, Fazel S. Abuse-deterrent extended-release oxycodone and risk of opioid-related harm. Addiction. 2021 Sep;116(9):2409-2415. doi: 10.1111/add.15392.
3. Harrison PJ, Luciano S. Incidence of Parkinson's disease, dementia, cerebrovascular disease and stroke in bipolar disorder compared to other psychiatric disorders: An electronic health records network study of 66 million people. Bipolar Disord. 2021 Aug;23(5):454-462. doi: 10.1111/bdi.13022.
4. Taquet M, Geddes JR, Husain M, Luciano S, Harrison PJ. 6-month neurological and psychiatric outcomes in 236 379 survivors of COVID-19: a retrospective cohort study using electronic health records. Lancet Psychiatry. 2021 May;8(5):416-427. doi: 10.1016/S2215-0366(21)00084-5.
5. Colbourne L, Luciano S, Harrison PJ. Onset and recurrence of psychiatric disorders associated with anti-hypertensive drug classes. Transl Psychiatry. 2021 May 26;11(1):319. doi: 10.1038/s41398-021-01444-1.
6. Taquet M, Luciano S, Geddes JR, Harrison PJ. Bidirectional associations between COVID-19 and psychiatric disorder: retrospective cohort studies of 62 354 COVID-19 cases in the USA. Lancet Psychiatry. 2021 Feb;8(2):130-140. doi: 10.1016/S2215-0366(20)30462-4.

**Table S1.** Medical codes used in defining the cohorts, in propensity score matching, and in defining the outcomes.

| Coding system and codes | Description |
| --- | --- |
| *ICD-10-CM* | *Diagnoses* |
| C00-D49 | Neoplasms |
| F10-F19 | Mental and behavioural disorders due to psychoactive substance use |
| F20-F29 | Schizophrenia, schizotypal, delusional, and other non-mood psychotic disorders |
| F30-F39 | Mood (affective) disorders |
| F40-F48 | Anxiety, dissociative, stress-related, somatoform and other nonpsychotic mental disorders |
| F51 | Sleep disorders not due to a substance or known physiological condition |
| F60-F69 | Disorders of adult personality and behaviour |
| F90-F98 | Behavioural and emotional disorders with onset usually occurring in childhood and adolescence |
| G44 | Other headache syndromes |
| G47 | Sleep disorders |
| G89 | Pain, not elsewhere classified |
| H04.12 | Dry eye syndrome |
| H10 | Conjunctivitis |
| J30 | Vasomotor and allergic rhinitis |
| J45 | Asthma |
| K13 | Other diseases of lip and oral mucosa (cheilitis) |
| L20-L30 | Dermatitis and eczema |
| L70 | Acne |
| L70.0 | Acne vulgaris |
| L70.1 | Acne conglobata |
| L70.8 | Other acne |
| L70.9 | Acne, unspecified |
| M25.5 | Pain in joint |
| M54 | Dorsalgia |
| M79.6 | Pain in limb, hand, foot, fingers and toes |
| R07 | Pain in throat and chest |
| R11 | Nausea and vomiting |
| R45 | Symptoms and signs involving emotional state |
| R45.0 | R45.0 Nervousness |
| R45.1 | R45.1 Restlessness and agitation |
| R45.2 | R45.2 Unhappiness |
| R45.3 | R45.3 Demoralization and apathy |
| R45.4 | R45.4 Irritability and anger |
| R45.5 | R45.5 Hostility |
| R45.6 | R45.6 Violent behaviour |
| R45.7 | R45.7 State of emotional shock and stress, unspecified |
| R45.8 | R45.8 Other symptoms and signs involving emotional state |
| R45.81 | R45.81 Low self-esteem |
| R45.82 | R45.82 Worries |
| R45.83 | R45.83 Excessive crying of child, adolescent or adult |
| R45.84 | R45.84 Anhedonia |
| R45.85 | R45.85 Homicidal and suicidal ideations |
| R45.850 | R45.850 Homicidal ideations |
| R45.851 | R45.851 Suicidal ideations |
| R45.86 | Emotional lability |
| R45.87 | Impulsiveness |
| R45.89 | Other symptoms and signs involving emotional state |
| R51 | Headache |
| R52 | Pain, unspecified |
| R53 | Malaise and fatigue |
| R68.2 | Dry mouth, unspecified |
| T14.91 | Suicide attempt |
| X71-X83 | Intentional self-harm |
| Y21-Y33 | Event of undetermined intent |
| *RxNorm* | *Medications* |
| 10753 (DE751) | Isotretinoin |
| AM200 | Erythromycins/macrolides |
| AM250 | Tetracyclines |
| CN300 | Sedatives and hypnotics |
| CN600 | Antidepressants |
| CN700 | Antipsychotics |
| HS051 | Glucocorticoids |

**Table S2.** Baseline characteristics of patients with acne in the isotretinoin and in the acne control (not exposed to any anti-acne agents, or oral antibiotics for acne) cohorts before and after matching.

|  | Before matching | | | After matching | | |
| --- | --- | --- | --- | --- | --- | --- |
| Percent | Isotretinoin (n=30866) | No selected anti-acne medicines (n=78666) | Std diff^a^ | Isotretinoin (n=28417) | No selected anti-acne medicines (n=28417) | Std diff^a^ |
| Mean age (SD) | 18.1 (3.5) | 18.2 (4.2) | 0.036 | 18.1 (3.5) | 18.1 (3.7) | 0.008 |
| Female | 46 | 62 | 0.328 | 47 | 47 | 0.015 |
| Black or African American | 4 | 10 | 0.223 | 4 | 4 | <0.001 |
| White | 75 | 65 | 0.209 | 74 | 73 | 0.007 |
| Mental and behavioral disorders due to psychoactive substance use | 2 | 2 | 0.038 | 2 | 2 | 0.013 |
| Schizophrenia, schizotypal, delusional, and other non-mood psychotic disorders | 0.2 | 0.3 | 0.026 | 0.2 | 0.2 | 0.008 |
| Mood (affective) disorders | 7 | 8 | 0.054 | 7 | 6 | 0.025 |
| Anxiety, dissociative, stress-related, somatoform and other nonpsychotic mental disorders | 10 | 11 | 0.037 | 9 | 8 | 0.035 |
| Sleep disorders not due to a substance or known physiological condition | 0.7 | 0.5 | 0.018 | 0.6 | 0.5 | 0.004 |
| Disorders of adult personality and behavior | 1 | 1 | 0.003 | 1 | 1 | 0.012 |
| Behavioral and emotional disorders with onset usually occurring in childhood and adolescence | 7 | 8 | 0.060 | 7 | 6 | 0.026 |
| Sleep disorders | 3 | 3 | 0.005 | 3 | 3 | 0.017 |
| Vasomotor and allergic rhinitis | 9 | 11 | 0.048 | 9 | 8 | 0.043 |
| Asthma | 7 | 8 | 0.027 | 7 | 7 | 0.024 |
| Dermatitis and eczema | 13 | 11 | 0.065 | 12 | 11 | 0.020 |
| Acne vulgaris | 92 | 71 | 0.561 | 91 | 92 | 0.008 |
| Acne conglobata | 50 | 42 | 0.166 | 50 | 50 | 0.004 |
| Acne, other | 52 | 46 | 0.134 | 52 | 52 | 0.016 |
| Acne, unspecified | 25 | 36 | 0.245 | 24 | 23 | 0.034 |
| Suicidal ideations | 1 | 1 | 0.009 | 1 | 1 | 0.013 |
| Suicide attempt | 0.1 | 0.1 | 0.008 | 0.1 | 0.1 | 0.008 |
| Intentional self-harm | 0.2 | 0.1 | 0.006 | 0.1 | 0.1 | 0.004 |
| Sedatives/hypnotics | 8 | 4 | 0.158 | 6 | 6 | 0.017 |
| Antidepressants | 12 | 6 | 0.199 | 10 | 9 | 0.020 |
| Antipsychotics | 2 | 1 | 0.070 | 2 | 2 | 0.013 |
| Glucocorticoids | 26 | 10 | 0.416 | 20 | 20 | 0.021 |

^1^Standardized mean difference. One patient can contribute to numbers in more than one diagnostic or medication group. Oral antibiotics: erythromycins(macrolides) or tetracyclines.

**Table S3.** Baseline characteristics of patients with acne in the isotretinoin and in the topical anti-acne agents cohorts before and after matching.

|  | Before matching | | | After matching | | |
| --- | --- | --- | --- | --- | --- | --- |
| Percent | Isotretinoin (n=30886) | Anti-acne agents, topical (n=108452) | Std diff^1^ | Isotretinoin (n=30460) | Anti-acne agents, topical (n=30460) | Std diff^1^ |
| Mean age (SD) | 18.1 (3.5) | 17.2 (4.2) | 0.226 | 18.0 (3.5) | 18.3 (3.9) | 0.061 |
| Female | 46 | 64 | 0.366 | 46 | 47 | 0.013 |
| Black or African American | 4 | 20 | 0.496 | 4 | 4 | 0.012 |
| White | 75 | 55 | 0.424 | 75 | 74 | 0.017 |
| Mental and behavioral disorders due to psychoactive substance use | 2 | 2 | 0.021 | 2 | 2 | 0.002 |
| Schizophrenia, schizotypal, delusional, and other non-mood psychotic disorders | 0.2 | 0.4 | 0.037 | 0.2 | 0.2 | <0.001 |
| Mood (affective) disorders | 7 | 9 | 0.074 | 7 | 7 | 0.004 |
| Anxiety, dissociative, stress-related, somatoform and other nonpsychotic mental disorders | 10 | 12 | 0.071 | 10 | 10 | 0.008 |
| Sleep disorders not due to a substance or known physiological condition | 0.7 | 0.7 | 0.008 | 0.7 | 0.7 | 0.001 |
| Disorders of adult personality and behavior | 1 | 2 | 0.079 | 1 | 1 | 0.003 |
| Behavioral and emotional disorders with onset usually occurring in childhood and adolescence | 7 | 11 | 0.137 | 7 | 6 | 0.024 |
| Sleep disorders | 3 | 5 | 0.069 | 3 | 3 | 0.005 |
| Vasomotor and allergic rhinitis | 9 | 15 | 0.162 | 9 | 9 | 0.029 |
| Asthma | 7 | 11 | 0.126 | 8 | 7 | 0.014 |
| Dermatitis and eczema | 13 | 18 | 0.145 | 13 | 13 | 0.010 |
| Acne vulgaris | 92 | 79 | 0.386 | 92 | 92 | 0.004 |
| Acne conglobata | 50 | 43 | 0.146 | 50 | 50 | 0.004 |
| Acne, other | 52 | 45 | 0.152 | 52 | 52 | <0.001 |
| Acne, unspecified | 25 | 31 | 0.129 | 24 | 24 | 0.022 |
| Suicidal ideations | 1 | 1 | 0.003 | 1 | 1 | 0.003 |
| Suicide attempt | 0.1 | 0.1 | 0.008 | 0.1 | 0.1 | 0.005 |
| Intentional self-harm | 0.2 | 0.1 | 0.003 | 0.2 | 0.1 | 0.008 |
| Sedatives/hypnotics | 8 | 7 | 0.050 | 8 | 8 | 0.005 |
| Antidepressants | 12 | 10 | 0.051 | 12 | 12 | 0.004 |
| Antipsychotics | 2 | 3 | 0.015 | 2 | 2 | 0.006 |
| Glucocorticoids | 26 | 23 | 0.075 | 25 | 25 | 0.011 |

^1^Standardized mean difference. One patient can contribute to numbers in more than one diagnostic or medication group. Oral antibiotics: erythromycins(macrolides) or tetracyclines.

**Table S4.** Baseline characteristics of patients with acne in the isotretinoin and in the oral antibiotics cohorts before and after matching.

|  | Before matching | | | After matching | | |
| --- | --- | --- | --- | --- | --- | --- |
| Percent | Isotretinoin (n=30866) | Oral antibiotics (n=44748) | Std diff^1^ | Isotretinoin (n=28398) | Oral antibiotics (n=28398) | Std diff^1^ |
| Mean age at index (SD) | 18.1 (3.5) | 18.0 (3.8) | 0.016 | 18.1 (3.5) | 18.1 (3.7) | 0.012 |
| Female | 46 | 58 | 0.236 | 50 | 50 | 0.001 |
| Black or African American | 4 | 8 | 0.157 | 4 | 5 | 0.006 |
| White | 75 | 72 | 0.072 | 74 | 74 | 0.001 |
| Mental and behavioural disorders due to psychoactive substance use | 2 | 3 | 0.103 | 2 | 2 | 0.007 |
| Schizophrenia, schizotypal, delusional, and other non-mood psychotic disorders | 0.2 | 0.5 | 0.052 | 0.2 | 0.3 | 0.010 |
| Mood (affective) disorders | 7 | 11 | 0.156 | 7 | 7 | 0.004 |
| Anxiety, dissociative, stress-related, somatoform and other nonpsychotic mental disorders | 10 | 15 | 0.144 | 11 | 10 | 0.003 |
| Sleep disorders not due to a substance or known physiological condition | 0.7 | 0.8 | 0.019 | 0.7 | 0.7 | 0.002 |
| Disorders of adult personality and behavior | 1 | 2 | 0.063 | 1 | 1 | 0.006 |
| Behavioural and emotional disorders with onset usually occurring in childhood and adolescence | 7 | 11 | 0.132 | 7 | 7 | 0.016 |
| Sleep disorders | 3 | 5 | 0.091 | 4 | 3 | 0.007 |
| Vasomotor and allergic rhinitis | 9 | 14 | 0.146 | 10 | 10 | 0.001 |
| Asthma | 7 | 11 | 0.123 | 8 | 8 | 0.002 |
| Dermatitis and eczema | 13 | 15 | 0.064 | 13 | 14 | 0.006 |
| Acne vulgaris | 92 | 83 | 0.270 | 91 | 91 | 0.011 |
| Acne conglobata | 50 | 51 | 0.025 | 51 | 51 | 0.002 |
| Acne, other | 52 | 53 | 0.011 | 54 | 53 | 0.006 |
| Acne, unspecified | 25 | 37 | 0.274 | 27 | 26 | 0.016 |
| Suicidal ideations | 1 | 2 | 0.053 | 1 | 1 | 0.004 |
| Suicide attempt | 0.1 | 0.1 | 0.014 | 0.1 | 0.1 | 0.002 |
| Intentional self-harm | 0.2 | 0.2 | 0.012 | 0.2 | 0.2 | 0.001 |
| Sedatives and hypnotics | 8 | 9 | 0.024 | 8 | 8 | 0.012 |
| Antidepressants | 12 | 16 | 0.112 | 13 | 12 | 0.011 |
| Antipsychotics | 2 | 4 | 0.071 | 2 | 2 | 0.009 |
| Glucocorticoids | 26 | 27 | 0.030 | 26 | 26 | 0.005 |

^1^Standardized mean difference. One patient can contribute to numbers in more than one diagnostic or medication group. Oral antibiotics: erythromycins(macrolides) or tetracyclines.

**Table S5**. Overall inpatient mortality among patients with acne exposed to isotretinoin compared with patient with dispensed prescriptions for oral antibiotics for acne who were not exposed to isotretinoin during follow-up by time after the index prescription.

| Time (months) | Isotretinoin (n=29629) | Oral antibiotics^1^ (n=29632) |  |  |  |  |  |
| --- | --- | --- | --- | --- | --- | --- | --- |
|  | Deaths | Deaths | OR | CI95% | Risk diff.^2^ (%) | CI95% | P-value |
| 3 | 10 | 10 | 1.00 | 0.42, 2.40 | 0 | -0.030, 0.030 | 0.999 |
| 6 | 10 | 10 | 1.00 | 0.42, 2.40 | 0 | -0.030, 0.030 | 0.999 |
| 9 | 14 | 10 | 1.40 | 0.62, 3.15 | 0.013 | -0.019, 0.046 | 0.414 |
| 12 | 19 | 12 | 1.58 | 0.77, 3.26 | 0.024 | -0.013, 0.060 | 0.208 |

Patients aged 12 – 27 years at index prescription in 2013 – 2019 after propensity score-matching for all covariates. Odds ratios (OR) and 95% confidence intervals (95%CI). ^1^Oral antibiotics: erythromycins(macrolides) or tetracyclines. ^2^Risk difference, 95% confidence interval (95%CI), and P-value for the risk difference.

Note: This further sensitivity analysis was not prespecified but conducted based on the peer-review comments. It was conducted on 17/01/2022 with an updated patient population. Compared to the original data, a larger number of patients was included in this analysis because more patients were included in the TriNetX platform.

**Table S6.** One-year incidence of neuropsychiatric outcomes among patients with acne compared with patients who were not diagnosed with acne.

|  | Acne diagnosis (n=382340) | | | No acne diagnosis (n=382340) | | |  |  |
| --- | --- | --- | --- | --- | --- | --- | --- | --- |
|  | Patients | Cases | IR/1000 | Patients | Cases | IR/1000 | OR | 95%CI |
| Any neuropsychiatric outcome | 286279 | 16417 | 57 | 343420 | 13725 | 40 | **1.46** | 1.43, 1.50 |
| Psychotic disorders | 380682 | 400 | 1 | 381239 | 448 | 1 | 0.89 | 0.78, 1.02 |
| Mood disorders | 345823 | 10072 | 29 | 367275 | 7041 | 19 | **1.53** | 1.49, 1.58 |
| Anxiety disorders | 334193 | 13331 | 40 | 365315 | 8637 | 24 | **1.72** | 1.70, 1.76 |
| Personality disorders | 374196 | 1328 | 3 | 380052 | 901 | 2 | **1.50** | 1.38, 1.63 |
| Behavioural disorders | 341865 | 4457 | 13 | 369040 | 3785 | 10 | **1.27** | 1.22, 1.33 |
| Sleep disorders | 362558 | 5340 | 15 | 376751 | 2789 | 7 | **2.00** | 1.91, 2.10 |
| Self-harm, non-fatal | 376587 | 1876 | 5 | 377003 | 1970 | 5 | 0.95 | 0.89, 1.01 |
| Dispensed prescriptions |  |  |  |  |  |  |  |  |
| Sedatives and hypnotics | 354173 | 8881 | 25 | 364345 | 11168 | 31 | **0.81** | 0.79, 0.84 |
| Antidepressants | 340364 | 11990 | 35 | 366775 | 8586 | 23 | **1.52** | 1.48, 1.57 |
| Antipsychotics | 372392 | 2558 | 7 | 376864 | 2719 | 7 | 0.95 | 0.90, 1.00 |
| Somatic symptoms^1^ | 245428 | 28858 | 117 | 298412 | 24996 | 84 | **1.46** | 1.43, 1.48 |
| Symtoms involving emotional state^2^ | 370942 | 3569 | 10 | 376380 | 2369 | 6 | **1.53** | 1.46, 1.62 |
| Inpatient visits^3^ | 382340 | 11876 | 31 | 382340 | 17903 | 47 | **0.65** | 0.64, 0.67 |
| Emergency visits^3^ | 382340 | 31001 | 81 | 382340 | 36364 | 95 | **0.84** | 0.83, 0.85 |

Patients aged 12 – 27 years at index healthcare visit in 2013 – 2019 after propensity score-matching for age at index visit, sex, and race. Incidence rate (IR) per 1000 person-years; Odds Ratios (OR) and 95% confidence intervals (95%CI). ^1^Somatic symptoms of common isotretinoin side effects, such as headache, nausea and vomiting, dry mouth, conjunctivitis, pain, cheilitis, and fatigue. ^2^Symptoms and signs involving emotional state, including various diagnoses for agitation and aggression. ^3^Including patients with visits before index prescription (before start of follow-up).

**Table S7.** One-year incidence of neuropsychiatric outcomes among patients with acne with dispensed prescriptions for topical anti-acne agents who were not exposed to isotretinoin or oral antibiotics for acne compared with patients with acne who were not exposed to isotretinoin, oral antibiotics for acne or topical anti-acne agents during follow-up.

|  | Topical anti-acne agents (n=70673) | | | No selected anti-acne medication^1^ (n=70673) | | |  |  |
| --- | --- | --- | --- | --- | --- | --- | --- | --- |
|  | Patients | Cases | IR/1000 | Patients | Cases | IR/1000 | OR | 95%CI |
| Any neuropsychiatric outcome | 55302 | 2742 | 49 | 54900 | 2367 | 43 | **1.16** | 1.09, 1.22 |
| Psychotic disorders | 70445 | 47 | 0.7 | 70426 | 55 | 0.8 | 0.85 | 0.58, 1.26 |
| Mood disorders | 65044 | 1633 | 25 | 64840 | 1375 | 21 | **1.19** | 1.10, 1.28 |
| Anxiety disorders | 63189 | 2212 | 35 | 62953 | 1855 | 29 | **1.19** | 1.12, 1.27 |
| Personality disorders | 69701 | 187 | 3 | 69653 | 181 | 2 | 1.03 | 0.84, 1.27 |
| Behavioural disorders | 64498 | 697 | 11 | 64363 | 627 | 10 | **1.11** | 1.00, 1.24 |
| Sleep disorders | 68146 | 806 | 12 | 68009 | 706 | 10 | **1.14** | 1.03, 1.26 |
| Self-harm, non-fatal | 69873 | 257 | 4 | 69737 | 252 | 4 | 1.02 | 0.85, 1.21 |
| Dispensed prescriptions |  |  |  |  |  |  |  |  |
| Sedatives and hypnotics | 67277 | 1447 | 21 | 67205 | 1070 | 16 | **1.36** | 1.25, 1.47 |
| Antidepressants | 65696 | 2077 | 32 | 65537 | 1420 | 22 | **1.47** | 1.38, 1.58 |
| Antipsychotics | 69614 | 393 | 6 | 69507 | 274 | 4 | **1.43** | 1.23, 1.67 |
| Somatic symptoms^2^ | 48607 | 4842 | 100 | 49937 | 4471 | 89 | **1.12** | 1.08, 1.17 |
| Symtoms involving emotional state^3^ | 69062 | 534 | 8 | 69143 | 441 | 6 | **1.21** | 1.07, 1.38 |
| Inpatient visits^4^ | 70673 | 1967 | 28 | 70673 | 1122 | 16 | **1.77** | 1.65, 1.91 |
| Emergency visits^4^ | 70673 | 4576 | 65 | 70673 | 3350 | 47 | **1.39** | 1.33, 1.46 |

Patients aged 12 – 27 years at index prescription (isotretinoin) or index visit (acne control group) in 2013 – 2019 after propensity score-matching for all covariates. Incidence rate (IR) per 1000 person-years; Odds ratios (OR) and 95% confidence intervals (95%CI). Oral antibiotics: erythromycins(macrolides) or tetracyclines. ^1^Healthcare contact during which a diagnosis for acne was recorded. ^2^Somatic symptoms of common isotretinoin side effects, such as headache, nausea and vomiting, dry mouth, conjunctivitis, pain, cheilitis, and fatigue. ^3^Symptoms and signs involving emotional state, including various diagnoses for agitation and aggression. ^4^Including patients with visits before index prescription (before start of follow-up).

**Table S8.** One-year incidence of neuropsychiatric outcomes among patients with acne with dispensed prescriptions for oral antibiotics for acne who were not exposed to isotretinoin compared with patients with acne with dispensed prescriptions for topical anti-acne agents who were not exposed to isotretinoin or oral antibiotics for acne during follow-up.

|  | Oral antibiotics^1^ (n=43878) | | | Topical anti-acne agents^2^ (n=43878) | | |  |  |
| --- | --- | --- | --- | --- | --- | --- | --- | --- |
|  | Patients | Cases | IR/1000 | Patients | Cases | IR/1000 | OR | 95%CI |
| Any neuropsychiatric outcome | 32321 | 1995 | 62 | 32758 | 1720 | 52 | **1.19** | 1.11, 1.27 |
| Psychotic disorders | 43654 | 47 | 1 | 43708 | 45 | 1 | 1.05 | 0.69, 1.57 |
| Mood disorders | 38898 | 1344 | 34 | 39252 | 1088 | 28 | **1.25** | 1.16, 1.36 |
| Anxiety disorders | 37548 | 1662 | 44 | 37996 | 1442 | 38 | **1.17** | 1.09, 1.26 |
| Personality disorders | 42943 | 163 | 4 | 43117 | 154 | 3 | 1.06 | 0.85, 1.32 |
| Behavioural disorders | 39152 | 525 | 13 | 39540 | 480 | 12 | 1.11 | 0.98, 1.25 |
| Sleep disorders | 41401 | 675 | 16 | 41721 | 579 | 14 | **1.18** | 1.05, 1.32 |
| Self-harm, non-fatal | 43021 | 232 | 5 | 43115 | 187 | 4 | **1.24** | 1.03, 1.51 |
| Dispensed prescriptions |  |  |  |  |  |  |  |  |
| Sedatives and hypnotics | 40062 | 1126 | 28 | 40361 | 985 | 24 | **1.16** | 1.06, 1.26 |
| Antidepressants | 37099 | 1469 | 39 | 37530 | 1150 | 31 | **1.30** | 1.21, 1.41 |
| Antipsychotics | 42332 | 388 | 9 | 42471 | 281 | 7 | **1.39** | 1.19, 1.62 |
| Somatic symptoms^3^ | 27166 | 3235 | 119 | 28393 | 2974 | 105 | **1.15** | 1.10, 1.22 |
| Symtoms involving emotional state^4^ | 42320 | 419 | 10 | 42480 | 371 | 9 | 1.13 | 0.99, 1.31 |
| Inpatient visits^5^ | 43878 | 1735 | 39 | 43878 | 1261 | 29 | **1.39** | 1.29, 1.50 |
| Emergency visits^5^ | 43878 | 4110 | 94 | 43878 | 3216 | 73 | **1.31** | 1.24, 1.37 |

Patients aged 12 – 27 years at index prescription in 2013 – 2019 after propensity score-matching for all covariates. Incidence rate (IR) per 1000 person-years; Odds ratios (OR) and 95% confidence intervals (95%CI). ^1^Oral antibiotics: erythromycins(macrolides) or tetracyclines. ^2^Topical antiacne agents, such as topical antibiotic preparations, tretinoin, and benzoyl peroxide. ^3^Somatic symptoms of common isotretinoin side effects, such as headache, nausea and vomiting, dry mouth, conjunctivitis, pain, cheilitis, and fatigue. ^4^Symptoms and signs involving emotional state, including various diagnoses for agitation and aggression. ^5^Including patients with visits before index prescription (before start of follow-up).
